# Nutritional Status in Community-Dwelling Elderly in France in Urban and Rural Areas

**DOI:** 10.1371/journal.pone.0105137

**Published:** 2014-08-18

**Authors:** Marion J. Torres, Béatrice Dorigny, Mirjam Kuhn, Claudine Berr, Pascale Barberger-Gateau, Luc Letenneur

**Affiliations:** 1 Univ. Bordeaux, ISPED, Centre INSERM U897-Epidémiologie-Biostatistique, F-33000, Bordeaux, France; 2 INSERM, ISPED, Centre INSERM U897-Epidémiologie-Biostatistique, F-33000, Bordeaux, France; 3 Nutricia Advanced Medical Nutrition, Danone Research, Saint-Ouen, France; 4 Nutricia Research, Advanced Medical Nutrition, Utrecht, Netherlands; 5 INSERM, U1061, Neuropsychiatrie: recherche épidémiologique et clinique, Université Montpellier I, Hôpital La Colombière, F-34093, Montpellier, France; University of Milan, Italy

## Abstract

Malnutrition is a frequent condition in elderly people, especially in nursing homes and geriatric wards. Its frequency is less well known among elderly living at home. The objective of this study was to describe the nutritional status evaluated by the Mini Nutritional Assessment (MNA) of elderly community-dwellers living in rural and urban areas in France and to investigate its associated factors.

**Methods:**

Subjects aged 65 years and over from the Approche Multidisciplinaire Intégrée (AMI) cohort (692 subjects living in a rural area) and the Three-City (3C) cohort (8,691 subjects living in three large urban zones) were included. A proxy version of the MNA was reconstructed using available data from the AMI cohort. Sensitivity and specificity were used to evaluate the agreement between the proxy version and the standard version in AMI. The proxy MNA was computed in both cohorts to evaluate the frequency of poor nutritional status. Factors associated with this state were investigated in each cohort separately.

**Results:**

In the rural sample, 38.0% were females and the mean age was 75.5 years. In the urban sample, 60.3% were females and the mean age was 74.1 years. Among subjects in living in the rural sample, 7.4% were in poor nutritional status while the proportion was 18.5% in the urban sample. Female gender, older age, being widowed, a low educational level, low income, low body mass index, being demented, having a depressive symptomatology, a loss of autonomy and an intake of more than 3 drugs appeared to be independently associated with poor nutritional status.

**Conclusion:**

Poor nutritional status was commonly observed among elderly people living at home in both rural and urban areas. The associated factors should be further considered for targeting particularly vulnerable individuals.

## Introduction

Worldwide, the proportion of elderly people is constantly increasing. According to the United Nations, in 2025, it is estimated that the population aged 60 years or older will be 1.2 billion and 2 billion in 2050 (representing about 22% of the world population) [1]. The risk of developing a chronic condition such as malnutrition increases with age [2]. According to the French National Authority for Health, malnutrition is caused by an imbalance between intake and the body's requirements. This imbalance causes tissue loss, in particular muscle tissue loss, with harmful functional consequences [3]. The potential risk factors of malnutrition are multiple: reduced food intake due to loss of appetite, episodes of fasting, poor dentition, swallowing difficulties, inability to eat independently, digestive disorders, chronic diseases and depression [3], [4]. Poor nutritional status is associated with higher risks of morbidity and mortality in elderly people [5] causing economic consequences for society [6].

The prevalence estimates of malnutrition in elderly are highly variable due to the use of different tools and different settings. In particular, there are few studies on malnutrition in community-dwelling elderly based on validated tools [5]. Moreover, individual characteristics that may influence the nutritional status of elderly community dwellers are poorly understood, such as living in rural or urban areas in the same country, which may influence lifestyle and food availability [7]. To determine nutritional status, the Mini-Nutritional Assessment (MNA) is one of the most recognised screening instruments and is used all around the world, especially in elderly people [3], [8], [9]. Since its first publication in 1996 [9], the MNA has been translated into more than twenty languages, including French. It has been validated with high sensitivity, specificity and reliability. It is an easy and cheap way to detect malnourished people or those at risk of malnutrition. The objective of this study was to describe the nutritional status of elderly community-dwellers, living in rural and urban areas in France, based on the MNA items, and to investigate its associated factors, notably socio-demographic factors, in order to better target individuals at risk [10].

## Methods

### Population and samples

For the current cross-sectional analysis, we used the baseline data of two French cohorts of elderly people aged 65 years and over: The AMI (Approche Multidisciplinaire Intégrée) cohort and Three-City (3C) Study.

Between 1999–2000, 9,294 elderly community-dwellers were included in the 3C cohort study, chosen from the electoral rolls of 3 large French cities and their suburbs: Bordeaux (n = 2,104), Dijon (n = 4,931) and Montpellier (n = 2,259). The aim of 3C is to study the vascular risk factors of dementia; its methodology was described previously [11].

In 2007, AMI included 1,002 subjects living in rural areas in Gironde, an administrative area in southwest France, randomly recruited from the reimbursement database of the unique French Farmer Health Insurance System. At baseline, 961 of these individuals were living at home. All had worked in the field of agriculture for at least 20 years. The aim of AMI is to study health and aging in elderly farmers living in rural areas. Details on this cohort have been published previously [12].

For 3C, the protocol was approved by the Consultative Committee for the Protection of Persons participating in Biomedical Research of the Kremlin-Bicêtre University Hospital (Paris). AMI was approved by the Ethics Committee of the University Hospital of Bordeaux according to the principles of the Declaration of Helsinki. All participants signed a written consent.

In both cohorts, data on socio-demographics, lifestyle, neuropsychological testing, physical examination, blood sampling, symptoms and complaints, medical history and food intake were collected at baseline.

### Mini Nutritional Assessment

The MNA is an 18-item questionnaire divided into four parts as described in Table 1 [9]: anthropometric measurements (i.e., weight, height, mid-arm circumference, calf circumference, and weight loss during the past 3 months); global assessments (six questions related to lifestyle, medication, and mobility); dietary questionnaire (eight questions related to number of meals, food and fluid intake, and autonomy of feeding); and subjective assessment (self-perception of health and nutrition). The aim of this tool is to identify elderly at risk of malnutrition or those who already are malnourished. A two-step procedure is applied to classify the subjects [13]. The first part of the questionnaire (items A to F) is administered and a score greater than 11 indicates a normal nutritional status. The second part of the questionnaire (item G to R) is administered to subjects with a score equal to or lower than 11. If the total score is greater or equal to 24, subjects are considered to have a normal nutritional status. A score between 17 and 23.5 indicates a risk of malnutrition and a score lower than 17 indicates a malnourished person. Due to the small number of subjects classified in the “malnutrition” category, the variable was dichotomised: “malnutrition” was collapsed with “at risk of malnutrition” to identify people in “poor nutritional status” versus those with a “normal nutritional status.”

**Table 1:** Correspondence between the items of the standard MNA and the proxy MNA in the AMI cohort.

Standard MNA	Proxy MNA
**Item A: Has food intake declined over the past 3 months due to loss of appetite, digestive problems, chewing or swallowing difficulties?**	**Item 2 of CESD: During the past week, I did not want to eat, my appetite was poor**
0) Severe decrease in food intake	0) Frequently, all the time
1) Moderate decrease in food intake	1) Never or very rarely, Occasionally
2) No decrease in food intake	2) Often
**Item B: Weight loss during the last 3 months**	**No proxy, same item.**
**Item C: Mobility**	**Restriction of mobility**
0) Bed or chair bound	0) Confined to bed
1) Able to get out of bed/chair but does not go out	1) Confined to home
2) Goes out	2) Confinement in close proximity, Confined to the quarter, Simple difficulty to use transport, No restrictions
**Item D: Has suffered psychological stress or acute disease in the past 3 months?**	**No proxy, same item.**
**Item E: Neuropsychological problems**	**Diagnosis of dementia, MMSE and CESD**
0) Severe dementia or depression	0) Diagnosis of dementia and MMSE<10, Depressive symptomatology by the CES-D
1) Mild dementia	1) Diagnosis of dementia and 10≥MMSE≤20 and CES-D negative
2) No psychological problems	2) Diagnosis of dementia and MMSE>20, CES-D negative
**Item F: BMI**	**No proxy, same item.**
**Item G: Lives independently**	**Scale of ADL of Katz**
0) No	0) Dependent on minimum one item
1) Yes	1) No dependence for each item
**Item H: Takes more than 3 prescription drugs per day**	**Listing of drugs taken according to the medical prescription**
0) No	0)≤3
1) Yes	1)>3
**Item I: Pressure sores or skin ulcers**	**No proxy, same item.**
**Item J: How many full meals does the patient eat daily?**	**No proxy, same item.**
**Item K: Selected consumption markers for protein intake? At least one serving of dairy products per day, Two or more servings of legumes or eggs per week, Meat, fish or poultry every day**	**FFQ for only dairy products, Do you eat dairy products per day?**
0) If 0 or 1 yes	0) No
0.5) If 2 yes	0.5) Yes but not > = 2 servings of legumes or eggs per week and not meat, fish or poultry every day
1) If 3 yes	1) Yes
	**No proxy for legumes, eggs, meat, fish and poultry consumption, same item**.
**Item L: Consumes two or more servings of fruit or vegetables per day?**	**FFQ: Do you eat fruits every day? Yes/No, How many times per day if yes, and per week if no? Same question with vegetables**
0) No	0) No
1) Yes	1) Yes, if they eat 1 fruit and 1 vegetable per day minimum or 2 fruits or 2 vegetables per day minimum
**Item M: How much fluid is consumed per day?**	**No proxy, same item.**
**Item N: Mode of feeding**	**Item 6 of ADL of Katz: Eating**
0) Unable to eat without assistance	0) Need help completely or artificial feeding
1) Self-fed with some difficulty	1) Need help to cook full meals
2) Self-fed without any problem	2) Need any help
**Item O: Self view of nutritional status**	**No proxy, same item.**
**Item P: In comparison with other people of the same age, how does the patient consider his/her health status?**	**No proxy, same item.**
**Item Q: Mid-arm circumference in cm**	**No proxy, same item.**
**Item R: Calf circumference in cm**	**No proxy, same item.**

Abbreviations: ADL = Activities Daily Living, BMI = Body Mass Index, CESD = Center for Epidemiologic Studies Depression Scale, FFQ = Food Frequency Questionnaire, MMSE = Mini Mental State Examination, MNA = Mini Nutritional Assessment.

### Reconstruction of the Mini Nutritional Assessment using proxy items

The MNA was administered in its standard version in the AMI cohort but was not included in the baseline questionnaire of the 3C cohort, which started 7 years earlier. However, some items of the MNA were also available in the 3C questionnaire, and other items could be replaced by similar questions that will be called proxy items. In the AMI cohort, these proxy items were also available. Therefore, a proxy MNA was constructed in the AMI cohort in order to assess its agreement with the standard MNA on the same subjects. The correspondence between the items of the standard MNA and the proxy MNA is given in Table 1. For item A regarding quantity of food intake, we used a question of the Center for Epidemiologic Studies Depression Scale (CES-D) [14] which is a scale used to identify depressive symptomatology. For item E on neurophysiological problems, we used the clinical diagnosis of dementia given by a neurologist combined with the score on the Mini Mental State Examination [15] to assess the severity of dementia (10 to 20 for a moderate dementia and 0 to 9 for a severe dementia). The CES-D scale was used to assess depressive symptomatology with a score superior or equal to 17 for men and superior or equal to 23 for women [16]. Item G on independency, was replaced by the Activity of Daily Living (ADL) scale developed by Katz [17] and subjects were considered independent if none of the ADL items was altered. Items about dairy products, fruits and vegetables consumption (items K and L) were replaced by the information obtained from a Food Frequency Questionnaire (FFQ) [18]. Item N about mode of feeding was replaced by a question from the ADL scale. In the construction of the proxy MNA, a lack of sensitivity was observed in the screening part of the questionnaire. Indeed, several subjects who scored 11 or less with the standard MNA and were therefore considered at risk of malnutrition, scored 12 with the proxy MNA and were classified as normal. In order to increase the sensitivity of the proxy MNA, the cut-off of the screening score was modified to 12 points or less for identifying individuals possibly at risk of malnutrition.

All the data needed to compute the proxy MNA were available for 692 subjects in AMI and 8,691 subjects in 3C.

### Socio-demographic information

Socio-demographic information included age (in 3 categories: <75 years, between 75 and 84 years and 85 years and older), gender, education (low level which represented no education or primary school only, medium level representing short secondary school: Certificate of Professional Aptitude (CAP) or the Diploma of Occupational Studies (BEP), and high level representing long secondary school: Baccalaureate degree or university), marital status (married, widowed and separated, single or other) and income (less than €750, €750 to €1,500, €1,500 to €2,250, more than €2,250 per month and refused to answer). In 3C, location (Bordeaux, Dijon or Montpellier) was also taken into account.

The autonomy of the subjects was assessed by the validated Katz ADL scale [17]. Individuals were considered to have a loss of autonomy when they presented at least one impairment in these five activities: bathing, dressing, toileting, transferring from bed to chair and eating.

### Statistical methods

Quantitative and qualitative variables were compared respectively by student t-test or chi-square test. Sensitivity, specificity and agreement of the proxy MNA were calculated using the standard MNA as the gold standard. Sensitivity was defined as the proportion of individuals correctly classified as having an impaired nutritional status. Specificity was defined as the proportion of individuals classified correctly as not having an impaired nutritional status. Agreement was assessed by the AC1 statistic [19] that showed less dependency upon trait prevalence [20] than the Kappa coefficient [21].

The proportion of subjects with a malnutrition status was estimated in both cohorts using the proxy MNA. As the proxy MNA showed different sensitivity and specificity than the standard MNA, the apparent frequency estimate was adjusted using the Rogan-Gladen estimator [22]. Statistical tests were performed at the 0.05 level of significance using the SAS statistical package (version 9.3; SAS Institute Inc., Cary, NC, USA).

## Results

Of the 961 subjects included in the AMI cohort and living at home, the standard MNA was available for 851 individuals. Excluded subjects had lower incomes, were more often demented (18% vs. 9%) and dependent for ADLs (11.0% vs. 5.0%). Among these 851 subjects, the proxy MNA was available for 692 individuals (81.3%). The 159 subjects with missing data in the proxy MNA were older, more often widowed, had a lower level of education, a lower income and were in poorer health.

The mean age of the 692 included subjects was 75.5 years (standard deviation (SD) 6.2). Participants were mainly males (62.0%), married (71.0%), had a low level of education (49.4% primary or less, 32.2% short secondary school) and half earned between 750 and 1,500 Euros per month (Table 2).

**Table 2:** Baseline description of the participants in the AMI and 3C cohorts.

	AMI (n = 692)	3C (n = 8,691)
	N (%)	N (%)
**Gender**		
Males	429 (62.0)	3,454 (39.7)
Females	263 (38.0)	5,237 (60.3)
**Age**		
65–74	346 (50.0)	5,155 (59.3)
75–84	295 (42.6)	3,141 (36.1)
≥85	51 (7.4)	395 (4.6)
**Marital status**		
Married	491 (71.0)	5,201 (59.9)
Widower	147 (21.2)	2,253 (25.9)
Single, divorced, separated or other	54 (7.8)	1,234 (14.2)
**Education**		
Low	342 (49.4)	2,219 (25.6)
Medium	223 (32.2)	3,098 (35.7)
High	127 (18.4)	3,365 (38.8)
**Income (Euros)**		
<750	64 (9.2)	458 (5.3)
750–1500	364 (52.6)	2,503 (28.8)
1500–2500	120 (17.3)	2,320 (26.7)
≥2500	46 (6.7)	2,874 (33.1)
Don't want to answer	98 (14.2)	536 (6.2)
**BMI (kg/m^2^)**		
≤21	15 (2.2)	916 (10.5)
21>BMI<25	155 (22.4)	3,234 (37.2)
25≥BMI<30	319 (46.1)	3,397 (39.1)
≥30	203 (29.3)	1,144 (13.2)
**Depressive symptoms**		
Yes	15 (2.2)	1,161 (13.4)
No	677 (97.8)	7,530 (86.6)
**Dementia**		
Yes	39 (5.6)	157 (1.8)
No	653 (94.4)	8,534 (98.2)
**Loss of autonomy (ADL)**		
Yes	17 (2.5)	78 (0.9)
No	675 (97.5)	8,598 (99.1)
**Using> 3 drugs**		
Yes	461 (66.9)	5,044 (58.0)
No	228 (33.1)	3,647 (42.0)

Abbreviations: 3C = Three-City study, ADL = Activities Daily Living, BMI = Body Mass Index.

Of the 9,294 subjects included in the 3C cohort, 603 were excluded due to missing data in the proxy MNA. Excluded subjects were more frequently women (67.5% vs. 60.3%), older, widowed (35.4% vs. 45.8%), less educated (no education or primary school level: 35.2% vs. 25.6%), had lower income and were in poorer health. The 8,691 remaining subjects had a mean age of 74.1 years (SD 5.5), were mainly females (60.3%), married (59.9%), had a medium level of education (35.7% short secondary school, 38.8% long secondary school and over), and had a medium level of income (59.8% earned more than 1,500 Euros) (Table 2).

Among the 692 subjects of AMI, the standard MNA identified 51 subjects with a poor nutritional status (7.4%, CI 95% 5.4–9.3). Using the proxy MNA, 44 subjects (6.4%, CI 95% 4.6–8.2) were identified as having poor nutritional status. The inter-rater reliability measured by the kappa coefficient showed good agreement (κ = 0.81) but was influenced by the low frequency of poor nutritional state; thus, instead we used the AC1 statistics that showed an excellent agreement with a value of 0.97. Using the standard MNA as the gold standard, the proxy MNA sensitivity was estimated to be 76.4% and the specificity to be 99.2%. Due to the imperfect characteristics of the proxy MNA, the Rogan-Gladen estimator was used and the corrected frequency of poor nutritional status was estimated to be 7.4%; hence, similar to that obtained with the standard MNA.

The proxy MNA was applied for the 8,691 subjects of the 3C cohort and 1,284 (14.8%) were identified as having poor nutritional status. The frequency of poor nutritional status using the Rogan-Gladen estimator was estimated to be 18.5%.

The characteristics associated with poor nutritional status were examined in each sample separately (Table 3). In AMI, older age, being widowed, a low BMI, being demented, having a depressive symptomatology, a loss of autonomy and an intake of more than 3 drugs appeared to be significantly associated with a poor nutritional status (p<0.05). In 3C, the similar trends were observed and female gender, a low education level and low income were also significantly associated with poor nutritional status in this cohort. In both cohorts, a low BMI was associated with poor nutritional status. However, poor nutritional status was also observed in overweight subjects (4.2% in AMI vs. 11.0% in 3C among individuals with a BMI greater than 25). The multivariate analyses included gender, marital status, level of education, level of income, BMI, depressive symptomatology (only in 3C), presence of dementia, loss of autonomy and intake of more than 3 drugs (Table 4). In AMI, low BMI, being demented and an intake of more than 3 drugs remained significantly associated with poor nutritional status. In 3C, female gender, marital status, BMI, depressive symptomatology, dementia, loss of autonomy and intake of more than 3 drugs remained significantly associated with poor nutritional status after controlling for other factors.

**Table 3:** Frequency of poor nutritional status evaluated by the proxy MNA according to baseline characteristics in the AMI and in 3C cohorts.

	AMI		p-value^1^	3C		p-value^1^
	n	Poor nutritional status (n = 44, 6.4%)		n	Poor nutritional status (n = 1284, 14.8%)	
**Gender**			0.17			<0.0001
Males	429	23 (5.4%)		3,454	338 (9.8%)	
Females	263	21 (8.0%)		5,237	946 (18.1%)	
**Age**			0.01			<0.0001
65–74	346	13 (3.8%)		5,155	640 (12.4%)	
75–84	295	25 (8.5%)		3,141	542 (17.3%)	
≥85	51	6 (11.8%)		395	102 (25.8%)	
**Marital status**			0.02			<0.0001
Married	491	23 (4.7%)		5,201	601 (11.6%)	
Widower	147	16 (10.9%)		2,253	469 (20.8%)	
Single, divorced, separated or other	54	5 (9.3%)		1,234	213 (17.3%)	
**Education**			0.53			<0.0001
Low	342	25 (7.3%)		2,219	375 (16.9%)	
Medium	223	11 (4.9%)		3,098	482 (15.6%)	
High	127	8 (6.3%)		3,365	423 (12.6%)	
**Income (Euros)**			0.25			<0.0001
<750	64	7 (10.9%)		458	115 (25.1%)	
750–1500	364	26 (7.1%)		2,503	458 (18.3%)	
1500–2500	120	5 (4.2%)		2,320	297 (12.8%)	
≥2500	46	3 (6.5%)		2,874	318 (11.1%)	
Don't want to answer	98	3 (3.1%)		536	96 (17.9%)	
**BMI (kg/m^2^)**			<0.0001			<0.0001
≤21	15	8 (53.3%)		916	395 (43.1%)	
21>BMI<25	155	14 (9.0%)		3,234	391 (12.1%)	
25≥BMI<30	319	17 (5.3%)		3,397	335 (9.9%)	
≥30	203	5 (2.5%)		1,144	163 (14.2%)	
**Depressive symptoms**			<0.0001			<0.0001
Yes	15	15 (100.0%)		1,161	667 (57.4%)	
No	677	29 (4.3%)		7,530	617 (8.2%)	
**Dementia**			<0.0001			<0.0001
Yes	39	9 (23.1%)		157	58 (36.9%)	
No	653	35 (5.4%)		8,534	1,226 (14.4%)	
**Loss of autonomy (ADL)**			<0.01			<0.0001
Yes	17	4 (23.5%)		78	42 (53.9%)	
No	675	40 (5.9%)		8,598	1,242 (14.4%)	
**Using> 3 drugs**			<0.001			<0.0001
Yes	461	41 (8.9%)		5,044	1,043 (20.7%)	
No	228	3 (1.3%)		3,647	241 (6.6%)	

Abbreviations: 3C = Three-City study, ADL = Activities Daily Living, BMI = Body Mass Index, MNA = Mini Nutritional Assessment.

1 Chi-square test.

**Table 4:** Factors associated with poor nutritional status in AMI and 3C cohorts: multivariate logistic regression analysis.

	AMI (n = 689)		p-value^1^	3C (n = 8664)		p-value^1^
	OR	CI 95%		OR	CI 95%	
**Gender**			0.71			<0.0001
Males	1			1		
Females	0.86	0.38–1.93		1.46	1.22–1.75	
**Age**			0.50			0.82
65–74	1			1		
75–84	1.61	0.70–3.67		0.98	0.84–1.15	
≥85	1.14	0.31–4.20		1.09	0.79–1.50	
**Marital status**			0.51			<0.01
Married	1			1		
Widower	1.69	0.66–4.33		1.36	1.12–1.66	
Single, divorced, separated or other	1.59	0.44–5.72		1.18	0.92–1.50	
**Education**			0.06			0.17
Low	1			1		
Medium	0.40	0.17–0.99		0.89	0.73–1.07	
High	1.41	0.53–3.73		0.82	0.66–1.01	
**Income (Euros)**			0.21			0.39
<750	1			1		
750–1500	0.73	0.26–2.06		0.84	0.61–1.14	
1500–2500	0.47	0.11–1.99		0.73	0.52–1.03	
≥2500	1.29	0.24–6.99		0.72	0.50–1.04	
Don't want to answer	0.15	0.02–0.89		0.83	0.55–1.26	
**BMI (kg/m^2^)**			<0.0001			<0.0001
≤21	23.09	5.10–104.46		9.11	7.39–11.23	
21>BMI<25	1			1		
25≥BMI<30	0.41	0.18–0.94		0.74	0.61–0.89	
≥30	0.16	0.05–0.50		0.96	0.75–1.22	
**Depressive symptoms**						<0.0001
No	NA		NA	1		
Yes	NA	NA		20.67	17.46–24.49	
**Dementia**			0.04			<0.0001
No	1			1		
Yes	3.04	1.08–8.57		3.42	2.22–5.28	
**Loss of autonomy (ADL)**			0.14			<0.0001
No	1			1		
Yes	3.38	0.68–16.74		6.94	3.91–12.31	
**Using>3 drugs**			<0.01			<0.0001
No	1			1		
Yes	10.40	2.59–41.69		3.52	2.95–4.20	

Abbreviations: 3C = Three-City study, ADL = Activities Daily Living, BMI = Body Mass Index, MNA = Mini Nutritional Assessment, NA = Not Available, OR = Odds Ratio.

1 Multivariate logistic regression including all variables presented in this table.

## Discussion

The frequency of poor nutritional status in elderly subjects living at home was estimated in two distinct samples and showed marked differences. The frequency was 7.4% in the rural sample (AMI) and was 18.5% in the urban sample (3C). Although the definition of malnutrition varies across studies, our results were similar to other studies in the community with a prevalence ranging from 7% to 17% [23]–[26]. A recent meta-analysis showed a prevalence of 37.7% for elderly people at risk of malnutrition or as being malnourished as evaluated by the MNA in community-dwellers [2]. The lower prevalence observed in our subjects may reflect the fact that our subjects were younger and had a higher BMI.

One of the interests of our study was to compare estimates of nutritional status in rural and urban areas. The frequency of malnourished people appeared to be more than twice as high in the urban sample. The different estimates between the two samples may be explained by the different composition of the cohorts. The AMI sample included more males and more often married subjects than the 3C sample, both characteristics associated with a lower risk of poor nutritional status. The AMI subjects have higher BMI. By contrast, participants of the AMI sample had a lower educational level and a lower income, both factors associated with a higher risk of poor nutritional status. Despite the fact that people in rural areas could have a more limited accessibility to shops and less accessible services related to nutrition because of longer distance to cover, this could be offset by greater solidarity between people and socialisation that could lead to higher food intake [27]. Indeed, elderly people in rural areas were more likely to be obese than those in urban areas [7]. This was also found in our samples and a lower BMI was associated with poor nutritional status after controlling for others factors. Moreover, the AMI sample is not fully representative of people living in a rural area but rather of people who worked in the agricultural sector. They may continue to produce food (eggs, chicken, vegetables …) and have a more diverse diet leading to better nutritional status.

The factors associated with poor nutritional status were in agreement with most of the recent studies conducted on malnutrition showing that older age [26], [28]–[30], gender (being female) [29]–[33], marital status (widowed) [28], [34], lower education [28], [35], lower income [5], [31], low BMI [26], [35], depressive symptoms [25], dementia [24], [32], loss of autonomy [32], [36] and polymedication [37] were associated with poorer nutritional status. In AMI, gender, marital status, level of education and income were not significantly associated with poor nutritional status, probably due to a lack of power, but these factors showed the same trends as the 3C sample.

The prevalence of malnutrition among elderly people is lacking in many studies because investigators did not included a specific tool to measure it, such as the MNA. However, the information to complete the MNA questionnaire was available. An alternative way to solve this problem could be to replace the missing information with other similar available data, as is the case in this study. The use of proxy variables to reconstruct the MNA was feasible and led to good agreement with the original tool. However, the estimations of the frequency of poor nutritional status are potentially under-estimated. First, participants in this analysis were selected since only subjects with no missing data were included. Indeed, in the AMI sample, the standard MNA was available on 851 subjects for whom the frequency of poor nutritional status was estimated to 9%. When considering only subjects with no missing data to reconstruct the proxy MNA, 692 subjects were included, leading to a frequency of poor nutritional status measured with the standard MNA of 7.4%. Secondly, the proxy MNA lacks sensitivity as it was estimated to be 76.4%, leading to an underestimation of the frequency of poor nutritional status, although the frequency was corrected using the Rogan-Gladen estimator. The lack of sensitivity is mainly due to border effects since subjects considered as having a poor nutritional status according to the standard MNA were close to the threshold when using the proxy MNA. One item (E: neuropsychological problems) is particularly sensitive to misclassification. Indeed, 13.4% of the individuals were considered to be without neuropsychological problems with the proxy MNA (according to the diagnosis of dementia and the MMSE for dementia and CES-D for depressive symptomatology) although they were considered to have moderate neuropsychological problems according to the standard MNA. For this reason, we decided to use another cut-off in the first part of the proxy MNA (increasing the threshold to 12 points) in order to get further information in the second part of the questionnaire and better classify the subjects. In the second part of the questionnaire, no item showed high discordance, but again, a border effect was observed. Indeed, among the 12 subjects considered to have a normal nutritional status with the proxy MNA and considered to have a poor nutritional status with the standard MNA, 8 subjects had a proxy MNA score equal to 24 or 24.5.

In conclusion, poor nutritional status was not uncommon in elderly people living at home in rural or urban areas in southwest France. Practitioners should monitor the nutritional status of their patients in order to participate in the reduction of the prevalence of this disorder and its consequences. Several factors are associated with poor nutritional state and practitioners should be encouraged to develop screening strategies according to these characteristics, even among subjects with a high BMI.
